# Smartphone-based educational and counseling interventions for women with high body mass index at Urmia’s health centers

**DOI:** 10.1186/s12905-023-02807-0

**Published:** 2024-01-02

**Authors:** Sayeh Ebrahimi Gangachin, Roghieh Bayrami, Bahlol Rahimi, Sima Masudi

**Affiliations:** 1 Graduate, Department of Midwifery, School of Nursing and Midwifery, Urmia University of Medical Sciences, Urmia, Iran; 2 Reproductive Health Research Center, Clinical Research Institute, Department of Midwifery, School of Nursing and Midwifery, Urmia University of Medical Sciences, Urmia, Iran; 3 Department of Health Information Technology, School of Allied Medical Sciences, Urmia University of Medical Sciences, Urmia, Iran; 4 Department of Epidemiology and Biostatistics, School of Medicine, Urmia University of Medical Science, Urmia, Iran

**Keywords:** Education, Counseling, Smartphone, Sedentary behavior, Fast food, Application

## Abstract

**Background and purpose:**

Overweight and obesity in women of reproductive age not only contribute to chronic diseases but also lead to fertility issues, adverse pregnancy outcomes, and psychological challenges. Among the detrimental behaviors associated with obesity, the consumption of fast foods and sedentary lifestyles have the most significant impact on weight gain and require effective interventions. This study aims to examine the effectiveness of an educational and counseling smartphone intervention in raising awareness and modifying behaviors related to sedentary behavior and fast food consumption in women with high body mass index.

**Materials and methods:**

This randomized controlled trial was conducted with two parallel groups comprising 106 women of reproductive age who sought assistance at Urmia health centers in 2022. Participants with diverse social and economic backgrounds were included. They were randomly assigned to either a control group or an intervention group. Valid and reliable questionnaires were administered to assess awareness regarding sedentary behaviors and fast foods consumption, as well as engagement in sedentary behaviors and consumption of fast foods. In addition to standard care, the intervention group received educational and counseling sessions through a dedicated smartphone application. Data analysis was performed using SPSS 20 software at a significance level of *P* < 0.05.

**Findings:**

The results of a statistical t-test indicated a noteworthy disparity between the control and intervention groups concerning the average awareness scores related to fast foods consumption (*P* < 0.001) and sedentary behaviors (P < 0.001) before and 3 months after the intervention. Additionally, a substantial statistical difference was observed in the mean consumption of fast foods (*P* < 0.001) and hours of sedentary behaviors (P < 0.001) before and 3 months after the intervention when comparing the two groups.

**Conclusion:**

Educational and counseling programs, when applied effectively, can serve the dual purpose of enhancing awareness concerning sedentary behaviors and fast foods consumption while concurrently fostering a reduction in the prevalence of these behaviors.

The study was registered in the Iranian Registry of Clinical Trials (IRCT Id: IRCT20210722051953N1) at 04/08/2021.

## Introduction

Over the past decade, sedentary behaviors have emerged as a significant public health concern [1, 2]. Sedentary behavior encompasses activities during waking hours that result in minimal energy expenditure while in a seated position, such as computer use, TV watching, leisure time, driving, lounging, or lying down [3]. Watching TV is among the most common daily sedentary behaviors [4]. A national survey conducted in Iran by the World Health Organization revealed that 76.3% of women and 58.8% of men aged 15–64 engage in sedentary behaviors, resulting in a total prevalence of 67.5% in this age group [5].

Research has shown a substantial increase in the association between sedentary behaviors and adverse health outcomes in recent decades [5]. Sedentary behavior contributes to 6% of cardiovascular diseases, 7% of type 2 diabetes cases, 10% of breast cancer deaths, and 10% of colon cancer cases, collectively contributing to 9% of premature deaths [6]. Previous meta-analyses have indicated that high levels of sedentary behaviors are linked to an increased risk of obesity and high blood pressure [7, 8]. Globally, women tend to be less active than men [6]. The detrimental impact of inactivity on health is particularly pronounced in individuals with a high body mass index (BMI), as they spend more time engaged in sedentary behaviors [9, 10].

Women of reproductive age who maintain a sedentary lifestyle are at risk of developing overweight and obesity [11]. These conditions not only lead to chronic diseases, but also pose risks during pregnancy, affecting fetal health and causing psychological distress for the mothers [12]. Pre-pregnancy overweight and excessive weight gain during pregnancy are associated with obesity, diabetes, high blood pressure, pre-eclampsia, post-delivery overweight, macrosomia, shoulder dystocia, and a higher rate of caesarean sections. Additionally, women with high BMIs are more prone to weak uterine contractions, leading to prolonged labor and the need for oxytocin to stimulate contractions [12].

Among the various unhealthy behaviors contributing to obesity, fast food consumption has the most significant impact [13, 14]. Fast foods are typically high in sugar, salt, fat, calories, and low in essential nutrients and fiber, often exceeding daily energy requirements [15]. Fast food consumption has surged globally in recent decades [16, 17]. Dadipour et al. (2013) found that fast food consumption is particularly high among teenagers, with 24% eating fast food more than once a week [18].

In today’s world, where healthcare costs are soaring, there is an increasing emphasis on shifting from a treatment-oriented approach to disease prevention. The World Health Organization highlights the importance of promoting health and adopting healthy lifestyles, creating supportive health environments, reorienting healthcare services, and formulating educational policies [19]. In health education, awareness plays a pivotal role in behavior change. Health promotion programs rely on the belief that altering awareness can lead to changes in attitudes and behaviors [20]. Evidence suggests that while short-term weight loss programs may yield initial positive results, sustaining these changes requires ongoing efforts. Thus, creating a conducive environment for individual change and employing specific behavioral therapy methods are crucial for obesity management [21, 22]. The World Health Organization advocates for the use of mobile health, incorporating mobile phones and personal digital devices in healthcare delivery [23]. Utilizing smartphone-based applications for delivering educational messages, audio, and video content to motivate and monitor patients’ health and treatment has proven effective [24]. The studies have demonstrated the success of smartphone-based educational programs in reducing sedentary behavior and promoting healthier habits [25, 26].

One challenge in practical health programs is the scarcity of scientifically sound educational content and the need for counseling [27]. This study aims to address these issues by providing education and counseling. Furthermore, during the COVID-19 pandemic, staying at home is a necessary measure to curb the virus’s spread. However, prolonged home confinement can lead to increased sedentary behaviors. In light of this, this study seeks to investigate the impact of educational and counseling interventions delivered through smartphones on awareness and behaviors related to sedentary behavior and fast food consumption among women with high body mass indexes who seek healthcare at comprehensive health service centers in Urmia in 2022.

## Materials and methodology

### Study setting

A randomized controlled trial (RCT) was conducted in Urmia, focusing on women of reproductive age with a high body mass index (BMI). The trial consisted of one educational intervention arm (smartphone app + routine care) and one control arm (routine care) for women who visited comprehensive health service centers in Urmia. The study design received approval from the Research Ethics Board of Urmia University of Medical Sciences (approval code: IR.UMSU.REC.1400.111).

### Eligibility and recruitment

To be eligible for the study, participants had to meet specific criteria, including being married women aged 15 to 49, having a BMI over 25, consuming fast food at least once a week, engaging in high sedentary behaviors, possessing at least reading and writing literacy (equivalent to 5th grade), not participating in educational programs related to physical activity in the past 6 months, not suffering from diseases related to physical activity, expressing willingness to participate, having no mental-psychological problems or sleep disorders, not taking slimming drugs, not following a special diet, lacking regular physical activity (such as walking at least three times a week for 30 minutes or not participating in sports clubs), having no specific diseases (e.g., diabetes, thyroid disorder, polycystic ovarian syndrome, cardiovascular issues, hypertension), possessing hearing and speaking abilities, and having a mobile phone with an Android system. Exclusion criteria included pregnancy during the research, experiencing unfortunate life events (e.g., death of loved ones, accidents resulting in disability), having medical problems, and unwillingness to continue cooperation.

### Randomization

**Fig. 1:**
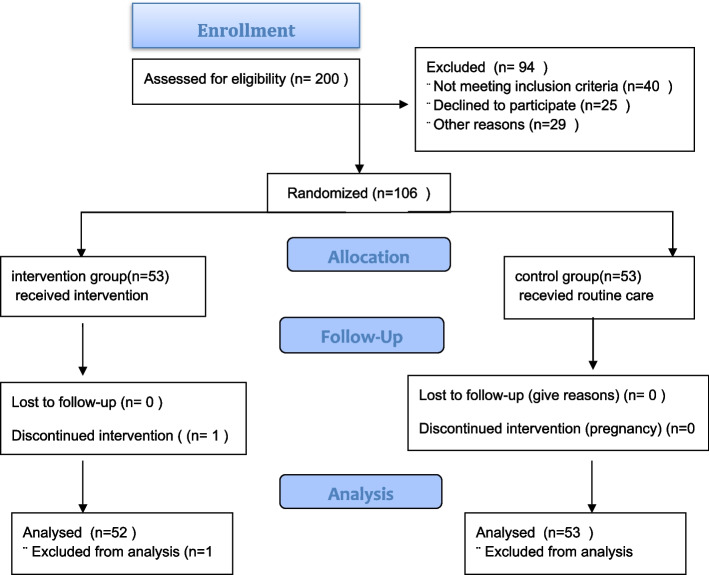
CONSORT diagram describing study enrolment and allocation

The determination of the requisite sample size for this study involved the utilization of the effect size, with careful consideration of a type 1 error rate of 0.05, an 80% power level, an effect size of 0.6, and a 20% anticipated attrition rate among participants during the study period. As a result, the total estimated sample size for this study was 106 individuals (refer to Fig. 1) and was determined accordingly [28]. The Comprehensive Health Service Centers within the Urmia region were stratified into three distinct categories based on their socio-economic status, as determined by experts from the Urmia Health Department. Specifically, 26 centers were categorized as having a high socio-economic and cultural status, 19 centers fell within the medium level, and 20 centers were designated as having a low socio-economic status. Within the context of this investigation, two centers were randomly selected from each stratum through a randomized lottery process. Even-numbered centers were allocated to the intervention group, while odd-numbered centers were assigned to the control group, resulting in a total of six study centers. Subsequently, the researcher accessed the Electronic Health System to compile a list of eligible women from these selected centers, assigning each woman a unique identifier. These identifiers were then transcribed onto small pieces of paper and placed within a receptacle. A third party, independent of the researcher, drew the slips from the receptacle in order to attain the desired sample size for each center, with the specific sample size contingent upon the center’s client population. Upon identification of potential participants, the researcher initiated telephone contact with each individual, seeking to secure their informed consent and motivation to partake in the research study. Subsequently, individuals from both the intervention and control groups were invited to their respective centers. In strict adherence to ethical principles, participants were presented with informed consent forms detailing their involvement in the research. The researcher provided assurance that all information divulged during counseling sessions and questionnaire responses would remain confidential.

### Description of the intervention

The designed version of the application program was installed on the mobile phones of the intervention group during the initial orientation session. Subsequently, comprehensive in-person instruction was provided on its utilization.

This application encompassed several core components, including user profiles containing demographic data (e.g., age, education, occupation, family income, number of pregnancies, live children, and abortions), as well as measurements for weight and height. Additionally, it featured educational materials pertaining to fast foods (comprising definitions and associated disadvantages), sedentary behavior (including explanations and adverse effects), and advisory resources, each represented by distinct icons. All educational content was centralized within the program, and users could access specific information by selecting the corresponding icons. Notably, the “body mass index” icon facilitated body mass calculations based on user-inputted height and weight in centimeters and kilograms. Educational materials concerning sedentary behavior were segmented into four sections, with one section activated for user engagement every other day during the initial week. This staggered approach ensured users’ sequential exposure to educational content. During the first week, users received two individual consultations. Throughout the three-month intervention period, users retained the ability to request advice within the application at their convenience. A similar framework was established for fast food-related content, which was introduced in the second week. Regular contact between the researcher and users, via phone or WhatsApp, ensured program engagement. Consultations encompassed discussions regarding factors influencing healthy dietary and physical activity behaviors, identification of behavior-changing obstacles, brainstorming solutions, reinforcement of correct behavioral patterns, self-confidence assessments vis-à-vis temptations, goal review, evaluation of barriers and enablers to healthy lifestyle behaviors from the participants’ perspective. Users also had the option to initiate selective counseling via WhatsApp or phone calls by accessing the counseling icon during the intervention. From the third week onward, daily reminders and recommendations regarding physical activity and fast food consumption were disseminated to the intervention group via the application at specified times. Sedentary behavior reminders were dispatched at 9 AM, 11 AM, 4 PM, 6 PM, and 9 PM. Concurrently, messages discouraging fast food consumption in favor of homemade alternatives were transmitted at 12 PM, 6 PM, and 9 PM. This messaging persisted for the duration of 3 months. In contrast, the control group received routine training provided by the comprehensive health service center, while both control and intervention groups continued to receive routine care from the comprehensive health centers. Three months after intervention, the health center conducted in-person evaluations of the control and intervention groups, encompassing assessments of fast food consumption, sedentary behaviors, and awareness levels related to sedentary behaviors and fast foods, and body mass index (BMI).

### Data collection tools

The data were gathered using a researcher–made questionnaire containing demographic and midwifery characteristics, awareness of fast foods and sedentary behaviors, a questionnaire for fast food consumption and Sedentary Behavior Questionnaire (SBQ) for adults [29]. Demographic and midwifery questionnaire contains personal information such as age, education, occupation, family income, weight, height, number of children, pregnancies, and abortions. Two separate questionnaires assessed awareness of fast foods (22 questions) and sedentary behaviors (16 questions) using response options: “correct,” “incorrect,” and “I don’t know,” with scores assigned accordingly. Scores ranged from 0 to 22 for fast foods awareness and 0 to 16 for sedentary behaviors awareness. Women’s fast food consumption was assessed by frequency of use in a past month and included 10 questions with response options: “never,” “once,” “twice,” “three times,” “four times,” “five times and more,” scored from 0 to 5. The sedentary behavior questionnaire measured daily time spent in 9 sedentary activities on weekdays and weekends including watching TV, playing computer games, listening to music while sitting, talking on the phone, doing office work, sitting and reading, playing a musical instrument, doing art, and driving/riding a car, bus, or train. These nine behaviors were completed separately for weekdays and weekend days. The participants answered this question: “On a normal weekday (or weekend day), how much time (from waking up to bedtime) do you spend on the following activities?” The options were “none”, “15 minutes or less”, “30 minutes”, “1 hour”, “2 hours”, “3 hours”, “4 hours”, “5 hours”, and “6 hours and more”. Sedentary hours were summed up separately for weekdays and weekends. Sedentary hours in all days were calculated using a balanced average: 7÷ (weekend hours × 2) + (weekday hours × 5) [29]. The baseline for sedentary behavior was 7 hours per day. The localization of the sedentary behavior questionnaire was done using the forward-backward method, and then its content validity and face validity were calculated and confirmed. The researcher made questionnaires were developed by the researchers. In order to check the validity of self-made questionnaires in this study, face validity and content validity were conducted using both qualitative and quantitative methods. The acceptable CVR rate according to the number of experts in the present study was 0.62 and above. In measuring the internal homogeneity, Cronbach’s alpha above 0.7 was acceptable.

### Data analysis

In this research, following data collection, we proceeded to input the data into SPSS software, version 20. To conduct a comprehensive data analysis, we employed various statistical measures. For qualitative variables, we utilized descriptive statistical indices such as number and relative frequency. In the case of quantitative variables with a normal distribution, we calculated the mean and standard deviation. Additionally, for quantitative variables with non-normal distribution, we employed the median and interquartile range to provide a thorough description of the data. Kolmogorov Smirnov statistical test was used to assess normal distribution of data. The independent t-test was used to compare inter-group differences, and Chi-square test was used to compare the categorical variables. Results were considered significant at *P* < 0.05.

## Findings

In this research, there were 53 participants assigned to the intervention group and 53 participants assigned to the control group. During the data analysis phase, one participant from the intervention group was excluded from the analysis due to incomplete questionnaire responses. The mean and standard deviation of the participants’ ages in both groups are presented in Table 1. A statistical comparison of the average age between the two groups indicated no significant difference (*P* < 0.001). Table 1 displays the individual, social, and qualitative characteristics of both groups. Utilizing the chi-square test, it was determined that there exists no statistically significant difference between the two groups in terms of the educational level of women and husbands, the employment status of women and husbands, and their economic status. In simpler terms, the two groups were found to be homogenous in these aspects. Table 1 provides insight into the obstetric characteristics of the two groups. Similarly, the chi-square test revealed no statistically significant difference between the groups in terms of the number of pregnancies, live children, abortions, and the history of infertility. Thus, it can be concluded that the two groups were also homogenous in these regards.

**Table 1:** Participants’ Socio demographic Characteristics (*n* = 105)

variables		Control group *n* = 53 Mean ± SD	Intervention group *n* = 52 Mean ± SD	*P*
**Age (year)**		29/4 ± 6/9	30/1 ± 7/0	^a^*p* = 0/615
		n (%)	n (%)	
**Education level of women**	Elementary	1 (2)	3 (6)	*P* = 0/884
	Middle school	5 (9)	4 (8)	
	High school and diploma	15 (28)	16 (31)	
	College education	32 (60)	29 (56)	
**Spouse’s level of education**	Elementary	0 (0)	1 (2)	^b^*p* = 0/793
	Middle school	5 (9)	5 (10)	
	High school and diploma	14 (26)	16 (31)	
	College education	34 (64)	30 (58)	
**Employment status of women**	Housewife	30 (57)	32 (62)	^b^*P* = 0/607
	Employee	23 (43)	20 (38)	
**Spouse’s employment status**	Private sector	28 (53)	25 (48)	^b^*P* = 0/791
	Employee	18 (34)	21 (40)	
	Laborer	1 (2)	4 (8)	
	Farmer	1 (2)	0 (0)	
	Other	5 (9)	2 (4)	
**Economic status of the family**	Inadequate	10 (20)	8 (15)	^b^*P* = 0/853
	Relatively adequate	31 (58)	33 (64)	
	Adequate	12 (22)	11 (21)	
**Number of pregnancy**	0	13 (25)	12 (23)	^b^*P* = 0/054
	1	15 (28)	22 (42)	
	2	15 (28)	12 (23)	
	3	9 (17)	3 (6)	
	4	1 (2)	3 (6)	
**Number of living children**	0	17 (32)	14 (27)	^b^*P* = 0/328
	1	18 (34)	25 (48)	
	2	14 (26)	10 (19)	
	3	4 (8)	2 (4)	
	4	0 (0)	1 (2)	
**Number of abortion**	0	38 (72)	42 (81)	^b^*P* = 0/275
	1	12 (22)	7 (13)	
	2	3 (6)	3 (6)	
**History of infertility**	yes	6 (11)	7 (13)	^b^*P* = 0/739
	No	47 (89)	45 (87)	

^a^Independent t test, ^b^Chi-square test of independence

## Efficacy of the intervention

### Impact of the mobile app on women’s awareness

The paired groups’ t-test results indicated a statistically significant difference in knowledge about fast food and sedentary behavior within the intervention group both before and after the educational intervention. Furthermore, the independent groups’ t-test revealed a statistically significant difference between the intervention and control groups after the educational intervention, as shown in Table 2.

**Table 2:** Comparison of the scores of awareness related to the consumption of fast foods and sedentary behaviors in control and intervention groups before and 3 months after the intervention

variables		Intervention group *n* = 52 Mean ± SD	Control group *n* = 53 Mean ± SD	*P*-value*
Awareness of consumption of fast foods	pre-intervention	12/87 ± 3/19	2/95 ± 13/79	0/125
	Post- intervention	20/65 ± 0/56	2/98 ± 13/85	< 0/001
Awareness of sedentary	pre-intervention	10/87 ± 3/02	2/29 ± 10/74	0/805
behaviors	Post-intervention	15/73 ± 0/56	2/31 ± 10/81	< 0/001

* Independent t test

### Impact of the mobile app on consumption of fast foods and sedentary behaviors

Additionally, the outcomes of the paired t-test have revealed a notable statistical distinction concerning the consumption of fast food and sedentary behavior within the intervention group, both prior to and following the educational intervention. Furthermore, subsequent to the educational intervention, a statistically significant divergence was observed between the intervention and control groups as depicted in Table 3.

**Table 3:** Comparison of the scores related to the consumption of fast foods and sedentary behaviors in the control and intervention groups before and 3 months after the intervention

variables		Intervention group *n* = 52 Mean ± SD	Control group *n* = 53 Mean ± SD	*P*- value*
consumption of fast foods	pre-Intervention	5/61 ± 1/43	1/48 ± 5/15	0/106
	Post- Intervention	0/42 ± 0/54	2/64 ± 4/3	< 0/001
sedentary behaviors	pre- intervention	9/39 ± 2/75	8/53 ± 2/64	0/106
	Post-intervention	4/21 ± 1/34	8/47 ± 2/61	< 0/001

*Independent t test

### Impact of the mobile app on women’s weight

The paired t-test results indicated a statistically significant variance in the weight of female participants within the intervention group, both before and after the educational intervention. Similarly, the t-test for independent groups demonstrated a statistically significant divergence between the intervention and control groups. The paired t-test results indicated a statistically significant variance in the weight of female participants within the intervention group, both before and after the educational intervention. Similarly, the t-test for independent groups demonstrated a statistically significant divergence between the intervention and control groups following the educational intervention, as illustrated in Table 4.

**Table 4:** Comparison of the average weight in two control and intervention groups before and 3 months after the intervention

	pre-intervention Mean ± SD	Post- intervention Mean ± SD	*P*-value*
**Intervention group** ***n*** **= 52**	77/8 ± 10/4	77/5 ± 10/3	< 0/001
**Control group** ***n*** **= 53**	79/4 ± 8/6	8/6 ± 79/4	

*Independent t test

## Discussion

This study aimed to investigate the impact of educational and counseling interventions via smartphones on awareness and behaviors related to sedentary behavior and fast food consumption in women with high body mass index. The findings revealed statistically significant differences in the average awareness scores related to sedentary behaviors after the intervention in both intervention groups compared to the control group. In consonance with this study, Compernolle et al. [30] conducted research that sought to evaluate the effects of mobile phone-based interventions on the aspects of interaction, acceptability, usability, and initial efficacy, particularly in self-monitoring for mitigating sedentary behavior in the elderly. Their findings suggest that the intervention was effective in heightening awareness, prompting individuals to become more cognizant of their sedentary behavior. Akinwusiet et al. [31] embarked on a study aimed at examining the influence of health education on the awareness of a sedentary lifestyle as a predisposing factor to cardiovascular diseases among secondary school principals in Nigeria, revealing a notable increase in participants’ awareness levels.

Additionally, the outcomes of this study have demonstrated that the intervention program significantly elevated awareness regarding the detrimental effects of fast-food consumption. Pertinent to this study, Manggabarani et al. [32] conducted research focusing on the impact of peer-based education on knowledge, attitude, and consumption of fast food among teenagers. The educational intervention had a marked effect on augmenting both attitude and knowledge levels pertaining to nutrition post-training. Similarly, Jorvand et al. [33] found that healthy nutrition education positively impacted knowledge, attitude, and behavior regarding fast food consumption. However, incongruent with our research, Mirkarimi et al. [34] conducted a study in Gorgan, which indicated that the educational program had no discernible impact on heightening awareness of healthy food among high school students concerning the factors affecting fast-food consumption. Likewise, Vahdaninia et al.’s study [35] focused on the effect of pamphlet-based education on fast-food consumption behavior among primary school students in Birjand, demonstrating that pamphlet education did not yield significant changes in students’ knowledge, attitudes, or behaviors in the domain of fast-food consumption. The use of readily accessible educational tools is increasingly seen as highly effective in conveying pivotal concepts that directly influence an individual’s health and overall quality of life.

Moreover, the results of the present study have evidenced that educational and counseling intervention via smartphone applications significantly reduced the duration of sedentary behavior among overweight women. In tandem with this study, Boerema et al. [36] conducted an intervention study to evaluate the potential impact and user experience of an mHealth intervention for mitigating sedentary behavior among older office workers. Participants in their study reduced their total sedentary time substantially and found the primary value of the intervention to lie in fostering awareness of their personal sedentary behavior patterns. Talebi et al. [37] embarked on a study to determine the influence of social networking through mobile phones in altering physical activity behavior in pregnant women. Their findings illustrated that education through social networks significantly increased the level of physical activity among pregnant women. Among the studies that are not in line with this study, the result of the study by Comprenol et al. [30] showed with the aim of investigating the effect of intervention through mobile phones in terms of factors, acceptability, usability and primary effectiveness for self-monitoring to reduce behavior. Elderly people are sedentary. It was investigated. The critical companies reported in relation to the intervention and mentioned it as motivation. They usually reported that the intervention changed elderly people thinking, and reducing their sedentary behavior.

The findings showed that there was a statistically significant difference in the average consumption of fast foods after the intervention in the intervention group and the control group. Among the studies in line with this study, we can refer to the results of the study by Yadav et al. [38] which was conducted with the aim of the effect of educational intervention on awareness, attitude and consumption of prepared foods among teenagers in India. Educational interventions led to a significant change in the awareness of the intervention group and their attitude towards the consumption of prepared foods had changed significantly and the number of students who used homemade foods had increased. Sheikh Ahmadi et al. [39] also conducted a study with the aim of the effect of an educational program based on the theory of planned behavior on reducing the consumption of fast foods in students of girls’ conservatories in Sanandaj city. After the intervention, the average of all studied structures in the intervention group was significantly higher than the control group. Among non-aligned studies, we can refer to the study by Fatehi Panah et al. [40], which aimed to investigate the effect of educational extension programs on the awareness, attitude and performance of female elementary school students regarding the consumption of fast foods, which the results indicated that education was not effective on performance and consumption of fast foods among students. Such a difference lies in the age of the study subjects and the difference in the intervention method.

In the present study, there was no statistically significant difference in the average weight of the women under study after the intervention in the intervention group and the control group. From the parallel study of Amini et al.’s study [41], a study was conducted with the aim of the effectiveness of the electronic training program on increasing the amount of physical activity and body mass index of female employees. The average score of body mass index after training in the intervention group did not show a significant change in comparison with the control group. One of the non-aligned studies was the one by Chen et al. [42], which measured the effect of a short-term smartphone-based intervention for overweight or obese Chinese-American adolescents. In the intervention group, in terms of weight, sugary drinks and time watching TV and computer and increased self-efficacy in nutrition and physical activity, it was significantly more than the control group. Weight loss had a significant relationship with reducing consumption of fast food and increasing physical activity. Also, a study by Khalili et al. [43] showed that using a mobile phone software called “step up” to measure obesity and overweight, monitor physical activity and count the number of steps of the women participating in the study has been effective. The reason for this difference may lie in the follow-up period, which in this study was 3 months, and BMI reduction usually occurs in 6 months. Considering that the family is also very important in the consumption of healthy foods and the physical activity of people as well as in the formation of personal habits and the health of people in childhood and in adulthood, providing education for a wide level of society and people’s friends and family in order to learn to transfer and inculcate the correct food and lifestyle in people in order to reduce the consumption of fast foods and more mobility and exercise and physical activity in subsequent studies through mobile phones is suggested. Among the limitations of the study was that due to the time limit, the follow-up of the samples for BMI changes was done 3 months after the intervention, which is recommended to be considered 6 months or more in other studies.

## Conclusion

Given the efficacy of utilizing smartphones as a means of instructing health-promoting behaviors, it is arguable that these mobile devices have functioned as catalysts for action and as reminders, leading to behavioral modifications. These changes encompass a reduction in sedentary activities and a decrease in fast-food consumption. It is noteworthy that the integration of contemporary technologies, including mobile applications, has proven invaluable during critical scenarios like the COVID-19 pandemic. This integration has enabled the surmounting of numerous challenges, including issues related to accessibility across diverse societal groups, the scarcity of service providers, and the excessive workload placed upon personnel.

## Data Availability

The data used and/or analyzed during the current study are available from the corresponding author on reasonable request.
